# Leber hereditary optic neuropathy: utilities and carer burden from British and Irish participants

**DOI:** 10.1186/s13023-025-03737-w

**Published:** 2025-05-07

**Authors:** Claire Lawrence, Emma Williams, Andrew Mumford, Steve Bojakowski, Julio Benedicto, Andrew Lloyd

**Affiliations:** 1 Acaster Lloyd, Lacon House, 84 Theobalds Rd, London, WC1X 8NL UK; 2 Trustee, LHON Society, The Hayloft, Pury Hill Business Park, Alderton, Northamptonshire, NN12 7LS UK; 3 Market Access Consultant, Bishop’s Stortford, UK; 4 GenSight Biologics S.A., 74, Rue de Faubourg Saint-Antoine, 75012 Paris, France

**Keywords:** Health related quality of life, Utilities, LEBER hereditary optic neuropathy, LHON, Visual acuity, Carer burden

## Abstract

**Background:**

Leber hereditary optic neuropathy (LHON) is a rare, maternally inherited, mitochondrial disease resulting in sudden, progressive central vision loss. The condition affects numerous aspects of daily life, functioning and overall health-related quality of life (HRQL), which may spillover to carers.

**Methods:**

Two studies were designed to estimate patient utilities associated with varying visual acuity in LHON (study 1) and to explore carer burden (study 2). In study 1, eight LHON health state vignettes (mild vision loss [LogMAR < 0.3] through to light perception [LogMAR ≥ 4]) were valued by the UK and Republic of Ireland (ROI) general pubic using the Health Utilities Index- 3 (HUI-3) and EQ-5D in an online survey (N = 360) and in time trade-off interviews (TTO; n = 120). In study 2, nine carers completed in-depth interviews exploring carer burden, the Care-related Quality of Life instrument (CarerQol), EQ-5D-5L and the Work Productivity and Activity Impairment Questionnaire (WPAI).

**Results:**

Study 1 demonstrated lower utilities for people with worse visual function. Mild vision loss (LogMAR < 0.3) was rated as 0.84 (HUI-3), 0.79 (EQ-5D) and 0.88 (TTO). Light perception (LogMAR ≥ 4), the most severe health state, was rated as 0.18 (HUI-3), 0.34 (EQ-5D), and 0.36 (TTO). In study 2, qualitative findings revealed substantial burden for many carers and family members. The most prominent impacts were emotional (e.g., guilt, devastation), especially related to the maternal inheritance of LHON. Impacts to carers’ daily life, social life and relationships, work, and finances were also described. Standardised measures identified little impact on HRQL (EQ-5D-5L = 0.89), but some carer related burden (CarerQol-7D = 78.4). The WPAI revealed an overall work impairment of 15% and activity impairment of 37%.

**Conclusions:**

Findings suggest the HUI-3 may be more sensitive to the HRQL impact of vision loss compared to the EQ-5D and TTO method. The data indicate the potential value of an effective treatment for LHON. Qualitative findings describe the impact of LHON on carers. However, the burden described in the qualitative data was incongruent with quantitative measures, particularly the EQ-5D-5L. This demonstrates the value of conducting mixed-methods research and the importance of selecting measures which capture population-relevant concepts.

**Supplementary Information:**

The online version contains supplementary material available at 10.1186/s13023-025-03737-w.

## Background

Leber hereditary optic neuropathy (LHON) is a rare, maternally inherited mitochondrial disease which leads to central vision loss due to optic neuropathy [1]. The condition most commonly presents in males aged 15–35 years but can occur at any age for both men and women [2]. Vision loss often starts with a visual defect in the central vision, typically in one eye at first, that progresses to blindness in both eyes [3]. The rate at which vision loss occurs varies; however around 80% of people with LHON are deemed legally blind within one year of symptom onset. As the disease progresses, colour vision and visual acuity in the rest of the visual field can also decline [3]. Central vision is used for tasks such as reading, driving, and recognising people and places. Therefore, LHON impacts many aspects of a person’s life including daily activities, emotional functioning, relationships, studies, work, recreation, and finances [4]. For many people, LHON can have a severe negative impact on quality of life and has been associated with a greater prevalence of smoking, excessive alcohol consumption and psychiatric comorbidities such as depression [5].

Novel gene therapies are being developed for the treatment of LHON. In a phase III trial, people with LHON treated bilaterally with one such therapy, lenadogene nolparvovec (GS010), demonstrated sustained and significant improvement in visual acuity versus natural history, with a favourable safety profile [6]. Novel treatments for conditions such as LHON require an assessment of benefit-risk by regulatory bodies and also an assessment of value which is undertaken often by Health Technology Assessment (HTA) authorities. Effective gene therapies such as eladocagene exuparvovec and voretigene neparvovec-rzyl are typically high-price treatments which have been shown to be cost-effective despite the high upfront cost. HTA review will typically include an assessment of the impact of the disease on patients’ health-related quality of life (HRQL). The impact on the wider family, particularly carers, can also be considered by HTA bodies.

In the UK, The National Institute for Health and Care Excellence (NICE) prefer utilities to be derived from the EQ-5D [7]. However, there is recognition that collecting EQ-5D data from patients may not be feasible, particularly in rare diseases, and that the EQ-5D may not be the most appropriate measure in some disease areas, such as vision loss [7, 8]. In such cases, alternative methods, such as the vignette approach, may be considered [9].

Two studies were designed to estimate patient utilities associated with varying levels of visual acuity in LHON (study 1) and to explore carer burden (study 2). In study 1, health state vignettes were developed to describe different levels of vision loss, using a combination of clinical trial data, literature review and in-depth qualitative interviews with clinical experts and people affected by LHON. The vignettes were then valued using two generic measures of HRQL: the EQ-5D-5L [10], the health utilities index (HUI-3) [11]. While both the EQ-5D-5L and HUI-3 are generic measures of HRQL, the HUI-3 includes a vision-specific domain. In addition, vignettes were valued using the time trade-off (TTO) valuation method.

In study 2, the impact of LHON on the wider family, particularly carers, was considered. In-depth qualitative interviews were conducted, supplemented by standardised measures of burden and HRQL including the Care-related Quality of Life instrument (CarerQol), EQ-5D-5L and the Work Productivity and Activity Impairment Questionnaire (WPAI).

## Study 1: patient utilities in LHON

Study 1 aimed to develop, validate and value patient health states in LHON.

### Method

#### Development of draft health state vignettes

Health state vignettes were defined by LogMAR boundaries in addition to three ‘off-chart’ states including counting fingers, hand motion and light perception. Eight health state vignettes were developed (Table 1). The definition of the health states was chosen to match the definitions in a cost-effectiveness model developed by the study sponsor (GenSight Biologics). Draft vignettes describing each health state were developed based on findings from a targeted literature review of quantitative and qualitative studies characterising the experience of living with LHON, previous vignettes for RPE65-mediated inherited retinal disease (IRD) [12] and Visual Function Questionnaire [13] (VFQ-25) data summarised by LogMAR group from the REFLECT trial [14].

**Table 1:** Definitions of the eight health states based on the Markov model structure

Health state	Definition LogMAR/ Snellen
*On-chart health states*	
HS1	LogMAR < 0.3 or 6/12
HS2	LogMAR ≥ 0.3 and < 0. or 6/24
HS3	LogMAR ≥ 0.6 and < 1.0 or 6/60
HS4	LogMAR ≥ 1.0 and < 1.3 or 6/126
HS5	LogMAR ≥ 1.3 and < 1.7 or 6/300
*Off-chart health states*	
HS6	Counting fingers (equivalent to LogMAR 2.0)
HS7	Hand motion (equivalent to LogMAR 2.3)
HS8	Light perception (equivalent to LogMAR 4.0)

Seven studies were identified in the targeted literature review which provided data to inform the description of the condition and impacts to HRQL. The seven studies resulted in the identification of three conceptual areas for inclusion in the health state vignettes: impacts to daily activities, social impacts, and emotional impacts [15–21].

**Fig. 1:**
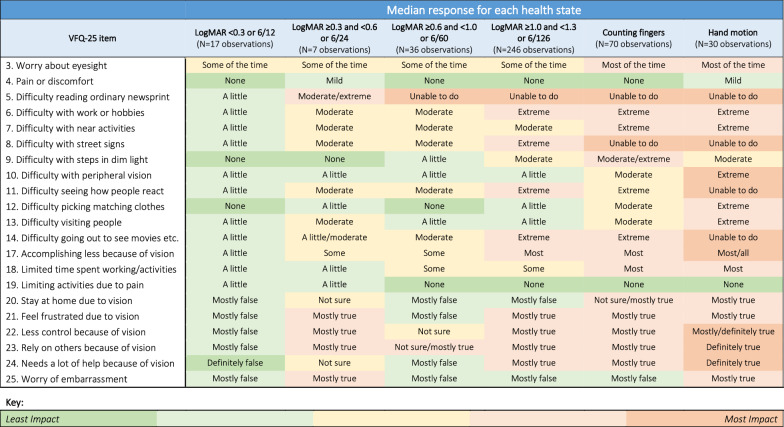
Summary of item-level VFQ-25 from the REFLECT trial

Patient reported outcome (PRO) data from the REFLECT clinical trial were only available for trial participants who met the visual function criteria for health states 1–4, 6 and 7. A summary of responses to 21 items from the VFQ-25 for these sub-groups provides a description of how vision related quality of life is different for people with different severities of vision loss (Fig. 1). These data directly informed the content of health vignettes. The data demonstrate that the impacts of LHON were greater for people with worse vision. Further, the data help differentiate between health states. For example, the data indicate that people typically worry about their eyesight some of the time in health states 1–4 but worry about their eyesight most of the time in health states 6 and 7.

The vignettes were drafted using lay language to ensure they were suitable for valuation by members of the general public.

#### Validation and finalisation of patient health states

Semi-structured qualitative interviews to understand the impact of LHON and validate the content of the draft health state vignettes were conducted with people with LHON as well as clinical experts. Semi-structured interviews follow a guide but allow the interviewer to explore issues that emerge during the interview in more detail. Interviews were conducted with participants in the UK and Republic of Ireland (ROI).

*Interviews with people with LHON*: Nine participants with LHON were recruited through patient advocacy groups (PAGs) in the UK (LHON Society) and ROI (LHON Society, Fighting Blindness; Supplementary File 1). Potential participants were asked to complete a brief screener questionnaire via email to ensure they met the inclusion criteria (adults with a self-reported diagnosis of LHON, resident in the UK or ROI). Interviews were scheduled with interested participants who provided consent verbally. Experienced qualitative interviewers followed a semi-structured interview guide. None of the participants were known to any of the interviewers.

The interviews were partly exploratory allowing participants to describe the impact of LHON in their own words. During the second part of the interviews, participants also provided feedback on the health state vignette that most closely matched their current level of visual acuity. Some participants also reviewed a second vignette depending on available time. Participants received a study remuneration of £50.

*Expert interviews*: Five clinical experts also completed a semi-structured interviews to review the vignettes, as well as fill in evidence gaps that were not addressed by the targeted literature review. Clinicians were presented with all health state vignettes on screen in sequence for review.

Feedback on draft health state vignettes were analysed using content analysis and used to develop the final set of health state vignettes (supplementary file). Changes that were made following feedback included specifying the impact of LHON on central versus peripheral vision, removing references to specific difficulties seeing at night-time and adding statements about ability to navigate familiar versus unfamiliar environments independently as well as the social difficulties of having a visual impairment that is not always obvious to others.

The final vignettes included eleven key concepts: description of the condition including the severity of visual impairment; ability to read words on a page, cross a street safely and recognise people; ability to see at different times of the day and go outside independently; ability to use electronic devices; ability to drive and use other modes of transport; emotional impacts; social impacts; ability to conduct usual activities; ability to participate in sport; pain experienced; and ability to participate in work or education (Supplementary File 2).

#### Valuation of health states

The vignettes were valued using an online survey and then subsequently in TTO interviews. This data collection was with members of the UK and ROI general populations. In the online survey, participants valued health states using two preference-based measures: the EQ-5D-5L and the HUI-3. A sub-sample of survey completers then valued the health states using the TTO method, which explores the willingness of participants to trade years of life for changes in quality of life [22]. (Supplementary Table 3 describes the different outcome measures used in this study).

*Valuation sample*: Members of the general public in the UK and the ROI were recruited via an online participant recruitment platform to take part in the online survey hosted via Qualtrics. Quotas were set to ensure an approximate match to the demographic profile of the general population (age, gender, ethnicity, country). All participants provided consent. The total sample (N = 362) included 301 participants from the UK and 61 participants from the ROI. A representative sub-sample of 120 survey completers (100 in the UK and 20 in the ROI) were recruited to participate in a follow-up TTO interview.

*Valuation procedures*: Participants completed a brief screener questionnaire and a background questionnaire including socio-demographic questions. Participants were asked to rate each vignette using the EQ-5D-5L and HUI-3, imagining they were living in the state of health described. To minimise participant burden, participants were randomised to value four (out of eight) health states, which were presented in a random order for each participant. Participants received a study remuneration of £5.25.

A representative sub-sample of survey completers (100 in the UK and 20 in the ROI) completed a follow-up TTO interview, valuing all health states. Trained TTO interviewers used a standard script to conduct the interviews. Interviews were conducted online due to COVID-19 restrictions. Participants received copies of the vignettes ahead of the interview. Participants were asked regularly if they could see the scale/board clearly, and to confirm that they understood the trade-offs they were being asked to make at every stage. Participants ranked the vignettes (plus a ‘dead’ state) against a VAS from 0 to 100, where 100 represents full health. In the TTO interview, participants were asked to imagine that they were living in the presented vignette. For each health state, they were asked to choose if they prefer either to live in the health state for 10 years followed by death or to live in [10–X] years of full health. The time in full health was varied using the ping-pong approach until they were indifferent between the choices. Participants received £40 for their participation in the interview.

#### Analyses

*Online survey valuations*: HUI-3 data were scored using the associated multiple attribute utility function [23]. EQ-5D-5L data were scored using the Hernandez et al. (2020) EQ-5D-5L to EQ-5D-3L mapping function [24]. EQ-5D VAS scores, and EQ-5D-5L and HUI-3 utility data were described using means and 95% confidence intervals (95% CIs).

*TTO valuations*: The TTO data were scored according to the point of indifference. The VAS ratings for each vignette were rescaled such that the value for the dead state was fixed at zero and all other values varied between 100 and the worse health state. The following formula was used to rescale the data.

$$V^{\prime } = \left( {\frac{{V - V_{Dead} }}{{100 - V_{Dead} }}} \right)*100$$

where *V’* is the rescaled VAS value, *V* is the original VAS value and ${V}_{Dead}$ is the value given to the Dead state. TTO and VAS scores were described using means and 95% CIs.

#### Ethical review

All study materials were submitted to the Western Copernicus Group Institutional Review Board (WCG IRB), a central IRB in the United States, for ethical review. The WCG IRB reviewed the documents and declared the study exempt from ethical review on 8th August 2022 (submission number: 1608918).

## Study 2: Carer burden and HRQL

Study 2 aimed to explore and quantify carer and family burden in LHON using a combination of in-depth qualitative interviews and standardised measures of burden and HRQL.

### Method

*Sample*: Qualitative interviews comprising semi-structured and structured questions were conducted with informal (unpaid) carers or family members of people with LHON residing in the UK and ROI. Nine participants carers were recruited through PAGs in the UK and ROI using recruitment adverts. Participants were informal (unpaid) carers or family member of someone with a self-reported diagnosis of LHON in the UK or ROI.

*Procedures*: Participants provided consent before each interview. During the interview, participants were asked to verbally complete a background questionnaire relating to themselves and the person they cared for. All interviews were conducted over Zoom or the phone using an interview guide containing semi-structured questions and standardised measures of burden and HRQL (EQ-5D-5L, CareQol-7D, WPAI). Interviews were conducted by experienced qualitative interviewers. Participants received a study remuneration of £50. All interviews were recorded and transcribed verbatim and de-identified for analysis purposes.

*Analyses*: Questionnaire data were scored according to published methods including the Hernandez et al. (2020) mapping function [24] [25] [26, 27] for EQ-5D. The semi-structured interview data regarding carer impacts (e.g., daily activities, physical, social, emotional, work, educational and financial impacts) were analysed using content analysis with MAXQDA [28] (www.maxqda.com), a software tool that assists with organizing qualitative data, but does not automate any of the analysis process. An initial coding framework was developed based on the interview guide. Transcripts were systematically coded against the coding framework, with new codes generated where necessary (i.e., where concepts outside of the interview guide were discussed by participants). Transcripts were independently coded by experienced qualitative researchers, and two of the transcripts were double coded by a second researcher to examine consistency.

#### Ethical review

All study materials were submitted to the WCG Institutional Review Board (WCG IRB), a central IRB in the United States, for ethical review. The WCG IRB reviewed the documents and declared the study exempt from ethical review on 8th August 2022 (submission number: 1608918).

## Results

### Valuation of health state vignettes

The vignettes were assessed using the HUI-3, EQ-5D-5L (n = 362) and through VAS ratings and the TTO interviews (total N = 120). Sample characteristics for the online survey and TTO sub-sample are presented in Tables 2 and 3.

**Table 2:** Online survey sample characteristics

	**Total sample (N=362)**		**UK sample (N=301)**		**ROI sample (N=61)**	
	**Mean**	**Range**	**Mean**	**Range**	**Mean**	**Range**
**Age**	46.47	18- 87	47.81	18- 87	39.89	20-74
	**Freq.**	**Percent**	**Freq.**	**Percent**	**Freq.**	**Percent**
**Gender**						
Female	183	50.6	152	50.5	31	50.8
Male	175	48.3	145	48.2	30	49.2
Identifies in another way	4	1.1	4	1.3	-	-
**Location**						
England	262	72.4	262	87.0	-	-
Scotland	26	7.2	26	8.6	-	-
Wales	7	1.9	7	2.3	-	-
Northern Ireland	6	1.7	6	2.0	-	-
Republic of Ireland	61	16.9	-	-	61	100
**Living situation**						
Living with partner/spouse	204	56.4	168	55.8	36	59.0
Living alone	84	23.2	79	26.3	5	8.2
Living with relatives	45	12.4	33	11.0	12	19.7
Other	29	8.0	21	7.0	8	13.1
**Employment**						
Employed, full-time	177	48.9	138	45.9	39	63.9
Employed, part-time	72	19.9	63	20.9	9	14.8
Retired	59	16.3	57	18.9	2	3.3
Unemployed	23	6.4	19	6.3	4	6.6
Student	13	3.6	8	2.7	5	8.2
Other	18	5.0	16	5.3	2	3.3
**Diagnosis of a chronic illness**						
No	221	61.1	180	59.8	41	67.2
Yes	141	39.0	121	40.2	20	32.8

**Table 3:** TTO sample characteristics

	**Total sample (N=120)**		**UK sample (N=100)**		**ROI sample (N=20)**	
	**Mean**	**Range**	**Mean**	**Range**	**Mean**	**Range**
Age	46.48	20-87	48.25	20-87	37.65	21-53
	**Freq.**	**Percent**	**Freq.**	**Percent**	**Freq.**	**Percent**
Gender						
Female	64	53.3	53	53.0	11	55.0
Male	55	45.8	46	36.0	9	45.0
Identifies in another way	1	0.8	1	1.0	-	-
Location						
England	87	72.5	87	87.0	-	-
Scotland	7	5.8	7	7.0	-	-
Wales	4	3.3	4	4.0	-	-
Northern Ireland	2	1.7	2	2.0	-	-
Republic of Ireland	20	16.7	-	-	20	100
Living situation						
Living with partner/spouse	61	50.8	49	49.0	12	60.0
Living alone	33	27.5	32	32.0	1	5.0
Living with relatives	12	10.0	10	10.0	2	10.0
Other	14	11.7	9	9.0	5	25.0
Employment						
Employed, full-time	62	51.7	49	49.0	13	65.0
Employed, part-time	25	20.8	22	22.0	3	15.0
Retired	17	14.2	17	17.0	-	-
Unemployed	5	4.2	4	4.0	1	5.0
Student	4	3.3	2	2.0	2	10.0
Other	7	5.8	6	6.0	1	5.0
Diagnosis of a chronic illness						
Yes	45	37.5	41	41.0	4	20.0
No	75	62.5	59	59.0	16	80.0

**Fig. 2:**
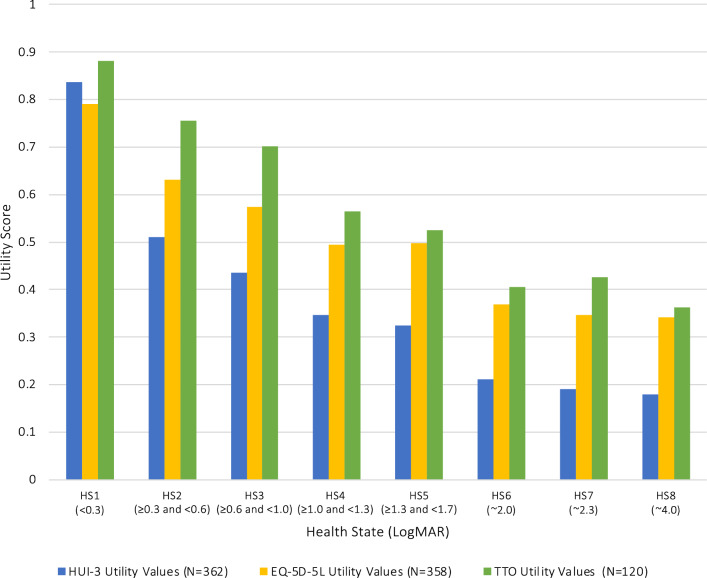
Comparison of HUI-3, EQ-5D-5L and TTO utility estimates

Generally, utilities were lower for states with lower visual acuity, and the lowest utility scores were observed for 7/8 health states using the HUI-3 (Table 4, Fig. 2). Mild vision loss (LogMAR < 0.3) was rated as 0.84 (HUI-3), 0.79 (EQ-5D) and 0.88 (TTO). Light perception (LogMAR ≥ 4), the most severe health state, was rated as 0.18 (HUI-3), 0.34 (EQ-5D), and 0.36 (TTO).

**Table 4:** Health state utilities based on HUI-3, EQ-5D-5L and TTO valuations of health state vignettes

	HUI-3 utilities mean (95% CI)			EQ-5D-5L utilities mean (95% CI)			TTO utilities mean (95% CI)		
Health state (LogMAR)	Total sample (N = 362)	UK sample (N = 301)	ROI sample (N = 61)	Total sample (N = 358)	UK sample (N = 297)	ROI sample (N = 61)	Total sample (N = 120)	UK sample (N = 100)	ROI sample (N = 20)
(< 0.3)	0.837 (0.808–0.866)	0.838 (0.807–0.870)	0.829 (0.755–0.902)	0.790 (0.770–0.810)	0.786 (0.763–0.809)	0.810 (0.776–0.844)	0.882 (0.859–0.905)	0.874 (0.847–0.901)	0.921 (0.888–0.954)
(≥ 0.3 and < 0.6)	0.511 (0.478–0.543)	0.504 (0.468–0.539)	0.548 (0.467–0.63)	0.632 (0.609–0.654)	0.625 (0.600–0.651)	0.663 (0.627–0.699)	0.756 (0.708–0.804)	0.746 (0.69–0.802)	0.806 (0.736–0.877)
(≥ 0.6 and < 1.0)	0.435 (0.404–0.466)	0.436 (0.404–0.469)	0.427 (0.339–0.514)	0.574 (0.549–0.600)	0.583 (0.555–0.611)	0.534 (0.471–0.596)	0.702 (0.65–0.754)	0.686 (0.625–0.746)	0.785 (0.709–0.861)
(≥ 1.0 and < 1.3)	0.347 (0.315–0.378)	0.351 (0.317–0.385)	0.324 (0.237–0.41)	0.495 (0.468–0.523)	0.506 (0.478–0.535)	0.439 (0.358–0.521)	0.565 (0.495–0.635)	0.546 (0.466–0.627)	0.658 (0.539–0.776)
(≥ 1.3 and < 1.7)	0.325 (0.295–0.354)	0.314 (0.281–0.346)	0.376 (0.305–0.447)	0.497 (0.469–0.525)	0.498 (0.468–0.528)	0.490 (0.414–0.566)	0.525 (0.448–0.602)	0.496 (0.408–0.584)	0.668 (0.539–0.796)
(~ 2.0)	0.211 (0.188–0.234)	0.212 (0.187–0.238)	0.204 (0.156–0.252)	0.368 (0.342–0.394)	0.373 (0.344–0.402)	0.343 (0.281–0.405)	0.406 (0.319–0.494)	0.391 (0.29–0.492)	0.485 (0.337–0.633)
(~ 2.3)	0.190 (0.164–0.216)	0.183 (0.153–0.212)	0.223 (0.163–0.283)	0.347 (0.315–0.378)	0.343 (0.307–0.378)	0.365 (0.295–0.434)	0.426 (0.348–0.504)	0.404 (0.316–0.493)	0.535 (0.397–0.673)
(~ 4.0)	0.180 (0.154–0.206)	0.177 (0.149–0.205)	0.193 (0.127–0.259)	0.341 (0.312–0.370)	0.339 (0.306–0.371)	0.354 (0.292–0.415)	0.363 (0.279–0.447)	0.342 (0.246–0.438)	0.469 (0.326–0.611)

There was some mis-ordering of health states, where a logically better health state (by visual acuity) state had a lower utility value than a ‘worse’ health state (EQ-5D-5L valuations of health states 4 and 5, TTO valuations of health states 6 and 7). None of these mis-orderings fell outside of the 95%CIs.

Generally, the HUI-3 produced lower utility estimates than the EQ-5D or TTO.

### Qualitative evaluation of family impact

Nine people participated in qualitative interviews to understand the impact of LHON on carers and family members. This included eight women who described caring for a partner/spouse, sibling or child with LHON. Some of the care recipients had received Idebenone and one had received a gene therapy. Sample characteristics are presented in Table 5.

**Table 5:** Sample characteristics of carers

	Total sample (N = 9)
	**Mean (range)**
Age	48.3 (30-70)
	**Freq. (Percent)**
Gender	
Female	8 (88.9%)
Male	1 (11.1%)
Relationship to care recipient	
Parent	5 (55.6)
Partner or spouse	3 (33.3)
Sibling	1 (11.1)
Carer LHON diagnosis	
Yes	0 (0%)
No	9 (100%)
	**Mean (Range)**
Age	32.0 (17-73)
Age at diagnosis	22.6 (9-58)
	**Freq. (Percent)**
Gender	
Female	3 (33.3)
Male	6 (66.7)
Treatment history	
Idebenone (private)	3 (33.3)
Idebenone (clinical trial)	3 (33.3)
GS010	1 (11.1)
Other	3 (33.3)
Carer reported severity of vision loss	
Very mild	0 (0.0)
Mild	0 (0.0)
Moderate	3 (33.3)
Severe	3 (33.3)
Very severe	3 (33.3)
Other health conditions	
Yes	3 (33.3)
No	6 (66.7%)

### Quantitative assessments of carer burden and HRQL

Carers’ mean EQ-5D-5L utility value was 0.893 (range 0.244 – 0.988, SD 0.245). The mean EQ-VAS score was 84.2 (range 72.5 – 95.0, SD 8.5). Many participants (7/9) responded that they had no problems in any of the EQ-5D-5L domains. The CarerQol-7D mean score was 78.4 (range 31.9 – 96.9, SD 21.0, median 85.9), where 0 represents the worst informal care situation and 100 represents the best informal care situation.

The majority of carers were in employment (N = 7, 78%). Mean percentage absenteeism in the past week due to caring responsibilities was 14% (range 0–100%) as assessed by WPAI. However, this value was driven by a single carer who was currently unable to work due to their circumstances. Of those in work, average presenteeism (impairment of work activities due to caring responsibility) was 15% (range 0–40%). Work productivity loss was also 15% on average (range 0–40%). Activity impairment was 36.7% (range 0–100%).

### Qualitative findings regarding carer burden

Two main themes are described: support provided and carer impacts. Each theme is presented below, characterised by relevant sub-themes and illustrated by participant quotes.

#### Theme 1: support provided

*Time spent caring*: The amount of time spent caring varied. Carers living in the same household as a person with LHON spent many hours providing support and care per day. As the time since diagnosis increased, the amount of support provided decreased as the person with LHON learnt to adapt to their vision loss and regain independence. A prominent theme to emerge from the interviews was carers’ constant availability to provide support to the person with LHON whenever required.

*“The rest of your time you're available or you're doing something so honestly it’s constant” –* CG05, parent

*Type of support*: Carers provided a wide range of support which could vary on a daily basis depending on the issue that arose or the person with LHON’s plans and social calendar. Carers provided support with daily activities including cooking, cleaning and selecting clothing as well as other administrative and household tasks. Carers also provided support with leisure activities such as exercise, hobbies and days out. Nearly all carers provided support with transport, such as driving and accompanying the person with LHON on public transport.

Carers provided social support, for example taking the person with LHON to meet their friends, as well as help with medical care such as organising travel and ordering medication.

Carers also described the emotional support they provided. At the time of diagnosis, carers helped the person with LHON to process and adjust to their vision loss while dealing with the impacts to their own emotional well-being. Carers also helped the person with LHON cope with the daily challenges and frustrations of living with the condition and often took the role of an understanding companion when they felt isolated. Although professional psychological support had been offered to most people with LHON, this was often not accessed, and instead most emotional support fell to carers. Some people with LHON were resistant to their carers attempts at providing emotional support and instead preferred to not discuss the impacts of their vision loss.

#### Theme 2: carer impacts

*Emotional impacts*: Carers reported feelings of devastation at the time of diagnosis and discussed the difficult adjustment period that followed. Carers who were mothers of a person with LHON discussed immense feelings of guilt for passing on the gene that caused their child’s vision loss. Worry about the future was a prominent theme across interviews. Carers’ fear and worry caused problems sleeping. Several carers felt stressed due to their responsibilities, which often resulted in feelings of emotional and physical exhaustion. The profound emotional impacts of caring for a person with LHON had knock-on effects to other areas of life. One carer was signed off work due to stress, whilst another went part-time to cope with the emotional demands of providing support.

*Impacts on daily life*: Many carers’ daily routines and activities changed to allow them to carry out their care tasks alongside their other responsibilities and commitments. Carers often prioritised the daily activities of the person with LHON over their own and consequently had more limited time to participate in leisure activities and hobbies. Carers often avoided planning holidays and trips and cancelled existing plans.

*“I have to spend more time at home than perhaps the average person would do, they’d get more involved in outdoor activities, clubs, societies, all that sort of thing, which is not really viable for me”* – CG04, partner/spouse

*Impacts on social life and relationships*: Some carers had more limited time to see friends due to providing support, while others intentionally isolated themselves to avoid discussing LHON with their friends. A lack of understanding about LHON among people in their social circles led some carers to cut ties with friends. In some cases, the physical and emotional demands of providing care had resulted in strained relationships with partners. Some carers felt bound by the person with LHON’s social calendar and obliged to accompany them to social events when the carers would have preferred to stay at home.

*“I haven’t been meeting up with friends properly since this started… I don’t want to sit and talk about it and I don’t want to not talk about it and sometimes they’ve not really been particularly understanding”* – CG01, parent

*Work and career*: Around the time of diagnosis most carers had taken time off work to support the person with LHON. One carer had been signed off work due to the emotional impacts of the diagnosis, while others had reduced their hours and work trips to cope with the logistical and emotional demands of providing care. Some carers reported that their care tasks could distract them from their work, especially when working from home with the person with LHON.

*“I’ve needed to be part time really, to have the time and emotional energy to deal with things that suddenly crop up and paperwork and dealing with meetings and so on”* – CG08, parent

*Wider family impacts*: Due to the genetic nature of the condition, family members often worried about themselves or their children losing vision in the future. In some cases, this fear became so pronounced that it caused some people to seek professional help to cope and in other cases prevented carers from having children. The disruption to family life made family activities that brought everyone together, such as watching films or playing boardgames, no longer viable.

*Positive impacts*: Despite many negative impacts, positive impacts of providing care were also reported. Carers were often proud of the person with LHON for how they adapted to their vision loss and their continued strength and determination to overcome daily challenges. Overcoming challenges together led some carers to have a stronger and closer relationship to the person with LHON, while others enjoyed the company and companionship that resulted from providing care. Carers often developed an increased awareness and understanding of disabilities.

*“I’ve seen him in such a strong light, like I said I’m so proud of him and I think it makes you have sympathy for other people that have got disabilities”* – CG06, parent

## Discussion

This project reports utilities for health states describing different levels of vision loss in people with LHON (study 1). An online survey and TTO interviews in the UK and ROI were used to derive utilities from health state vignettes. The use of vignettes to capture HRQL data for economic evaluation is common, especially in rare diseases [29]. In the current project, different sources of information were used to develop eight vignettes which described symptoms, functioning and HRQL for a typical patient in each health state. As expected, the highest utilities were found for health state 1 (LogMAR < 0.3) and the lowest utilities were observed for health state 8 (light perception). The HUI-3 scores were lower than the EQ-5D-5L scores, which is likely due to the inclusion of a domain specifically related to vision in the HUI-3.

The content of the vignettes describing the impact of LHON on quality of life determines in large part the utility results. The quality of the results thus depend on the accuracy of the vignettes. For this reason, different sources of data were brought together to inform the content. The variety of data sources combined with iterative rounds of review helped ensure that the vignettes were balanced, representative and accurate.

In addition, the vignettes were heavily informed by clinical trial data in people with LHON. Response data from the VFQ-25 were summarised by level of visual acuity (in line with health state definitions). This meant that some statements in the vignettes were drawn directly from the validated VFQ-25 items, reflecting the actual experience of trial participants in a given health state. However, VFQ-25 data were not available for health states 5 and 8, so some vignettes were developed without this type of insight. To compensate for this gap, detailed qualitative work was undertaken with people with LHON and clinicians to refine the content of the vignettes.

The utility weights from the present study can be compared to published data from other studies describing vision loss, but this comparison is limited by the fact that none of these studies involved people with LHON. Nonetheless, such a comparison could serve to verify directionally the validity of the utility weights. The range of utilities (HUI-3 derived) in the present study was 0.84 (LogMAR < 0.3) to 0.18 (Light perception), a difference of 0.66. Previous research has identified a similar wide range of scores – 0.65 (20/200 to 20/400) to 0.26 (no light perception) from self-evaluation of health using TTO in a group of people with different visual disorders [30].

Another study [31] presented EQ-5D data from people with diabetic retinopathy, which reported vision loss at 6/6 to 6/9 had a utility score of 0.75, and counting fingers/hand motion was 0.34. The different clinical context needs to be taken into account, because quality of life is not solely driven by visual acuity. People with macular oedema or diabetic retinopathy have a different age and comorbidity profile to people with LHON. In a condition like diabetic retinopathy, we may expect people whose vision is off-chart would have worse HRQL (compared with LHON) because they are likely to also have other diabetic complications. Despite these limitations, previous research consistently shows that poor levels of visual acuity have a substantial effect on HRQL [19].

This study used several different measures for assessing HRQL in the context of LHON. The EQ-5D-5L was included because it is perhaps the most widely used method for estimating utility generally and preferred by national bodies like NICE. However the EQ-5D does not specifically measure vision, which is an important limitation in this context. Like the EQ-5D-5L, the HUI-3 is designed to be used for the estimation of utilities for cost effectiveness research, but unlike the EQ-5D-5L, it does specifically assess vision loss. Figure 2 shows a greater differentiation between health states when measured by HUI-3 than EQ-5D – this is probably due to the specific inclusion of vision loss. In a clinical trial context, then, the HUI-3 may be more appropriate as a measure of HRQL in vision disorders. The third method that was used to evaluate the vignettes was the TTO. The TTO evaluation produced higher scores than the two standardized measures, which is a difference that could have an important impact on an economic evaluation. From these findings we suggest that the best solution is to capture HRQL data directly from LHON patients in each state. And the participants should probably be assessed using the HUI-3.

This project also reports the impact of LHON on informal carers and family members (study 2). Carer and family burden was explored through interviews in which three well-validated questionnaires were administered and the type of support provided, as well as the impacts of providing support, were discussed. The results from the questionnaires suggest a relatively small burden to carers with minimal impacts to HRQL, work and regular activities reported – which is somewhat surprising For example, the mean EQ-5D utility was 0.89, compared with a UK population average of 0.85 in adults aged 45–54 [32].

The quantitative measures, however, may be underestimating the impact on carers, as the qualitative data reveal a more pronounced effect on carers. These data described a substantial emotional impact, as well as impacts on daily life, social impacts, and impacts to work. Carers supported a wide range of activities, and while the reported time actually spent caring show a wide variation, participants uniformly described the stress from being constantly ‘on call’ to provide support. Impacts extended beyond the primary carer, with other family members being involved in providing support, and the diagnosis of LHON having profound consequences (both emotional and practical) for relatives. These findings are consistent with other research exploring carer burden in LHON [33].

Limitations of the project should be considered when interpreting the results. Despite the use of different data sources it is worth considering that the vignettes are inevitably a simplification. They cannot capture the range of different experiences and impacts that people experience. The vignettes were reviewed during interviews with people with LHON, but recent visual function assessments were not available for these participants and so they were assigned a vignette to review based only on self-reported visual function, rather than actual visual acuity. Therefore, there is some uncertainty about whether participants were reviewing the health state that matched their current level of visual acuity.

Generally, the utilities were lower for vignettes that described worse visual acuity. However, there was a small amount of ‘mis-ordering’ of health states, where an objectively better health state (in terms of visual acuity) had a lower utility value than a worse health state. None of these mis-orderings fell outside of the 95%CIs and so could be considered just measurement error.

Finally, it’s also worth noting that members of the general public were required to imagine living in the health state described when making valuations. There is uncertainty around how feasible it is for people to imagine living with a visual impairment despite the information provided.

## Conclusions

This research demonstrates the substantial burden associated with LHON, and potential spillover effects on carers and family members. The findings highlight the unmet need for treatments for this condition and identify opportunities for improving the instruments used to assess the quality of life impact of the disease and potential treatments.

## Supplementary Information

Additional file1 (DOCX 34 KB)

Additional file2 (DOCX 38 KB)

Additional file3 (DOCX 17 KB)

## Data Availability

Data and materials are available upon reasonable request to the corresponding author.

## Abbreviations

LHON: Leber hereditary optic neuropathy
HTA: Health technology assessment
HRQL: Health-related quality of life
NICE: National institute for health and care excellence
TTO: Time trade-off
CarerQol: Care-related quality of life instrument
WPAI: Work productivity and activity impairment questionnaire
IRD: Inherited retinal disease
VFQ-25: Visual function questionnaire
PRO: Patient reported outcome
PAGs: Patient advocacy groups
UK: United Kingdom
ROI: Republic of Ireland
VAS: Visual analogue scale
WCG-IRB: Western Copernicus group institutional review board
HUI: Health utilities index
