# Supplementary material for: Leber hereditary optic neuropathy: utilities and carer burden from British and Irish participants
Source: Orphanet J Rare Dis. 2025 May 7;20:219. doi: 10.1186/s13023-025-03737-w (PMC12060539; doi:10.1186/s13023-025-03737-w)
Supplement: Supplementary file 1 — Additional file1 (DOCX 34 KB) [file 13023_2025_3737_MOESM1_ESM.docx]

**Supplementary file 1: Sample characteristics for people with LHON**

|  | Mean (range) |
| --- | --- |
| Age | 45.2 (28-76) |
| Age at diagnosis | 35. 9 (15-69) |
|  | **Freq. (Percent)** |
| Gender |  |
| Female | 1 (11.1) |
| Male | 8 (88.9) |
| Mutation type |  |
| ND4 | 7 (77.8) |
| Other | 2 (22.2) |
| Treatment history |  |
| Idebenone (private) | 5 (55.6) |
| Idebenone (clinical trial) | 1 (11.1) |
| Idebenone (named patient scheme) | 1 (11.1) |
| GS010 | 2 (22.2) |
| Other | 1 (11.1) |
| Self reported severity of vision loss |  |
| Very mild | 0 (0.0) |
| Mild | 2 (22.2) |
| Moderate | 1 (11.1) |
| Severe | 4 (44.4) |
| Very severe | 2 (22.2) |
| Other health conditions |  |
| Yes | 3 (33.3) |
| No | 6 (66.7) |
