# Supplementary material for: Leber hereditary optic neuropathy: utilities and carer burden from British and Irish participants
Source: Orphanet J Rare Dis. 2025 May 7;20:219. doi: 10.1186/s13023-025-03737-w (PMC12060539; doi:10.1186/s13023-025-03737-w)
Supplement: Supplementary file 2 — Additional file2 (DOCX 38 KB) [file 13023_2025_3737_MOESM2_ESM.docx]

**Supplementary file 2: Final health state vignettes**

**HS1 – LogMAR <0.3 or 20/40**

- You have an eye condition that has caused a mild impairment of your central vision. You have some use of your peripheral vision. You have been told that your vision may continue to deteriorate.
- You can read words on a page with minimal difficulty. You can usually recognise people when you meet them.
- You are able to use mobile phones, tablets and computers with minimal or no adaptation.
- You can navigate familiar and unfamiliar environments independently with minimal difficulty.
- You are able to drive. However, you may find driving a little difficult and may not feel confident because of your visual impairment.
- Your vision loss can be very difficult to come to terms with. You sometimes feel frustrated and worry about the future. You sometimes feel anxious or depressed.
- You can mostly socialise normally and are able to meet new people. You are able to go to social events such as restaurants.
- You are able to conduct most of your usual activities (e.g. shopping, cooking and paperwork) without help from others.
- You can physically do most sports, such as gym work, with minimal assistance. Your vision loss limits some of the types of sports you can take part in.
- Your eye condition does not cause you any pain.
- You are able to participate in work or education with minimal assistance. Your condition means that some career paths and roles may not be an option for you.

**HS2 – LogMAR ≥0.3 and <0.6 or 20/40 to 20/80**

- You have an eye condition that has caused a mild to moderate impairment of your central vision. You have some use of your peripheral vision. You have been told that your vision may continue to deteriorate.
- You can read words on a page with moderate difficulty and may require a visual aid (e.g. magnification). You sometimes struggle to recognise people and may rely on a person’s shape/hair colour or voice in order to recognise them.
- You are able to use mobile phones, tablets and computers but may require some adaptation (e.g. magnification)~~.~~
- You can mostly navigate familiar environments with minimal difficulty. You have some difficulty navigating unfamiliar environments independently.
- You are unable to drive and get limited use of public transport. Your visual impairment means that you rely on friends, family members or taxis to take you where you need to go.
- Your vision loss can be very difficult to come to terms with. You sometimes feel frustrated and worry about the future. You sometimes feel anxious or depressed.
- You sometimes find socialising difficult. You often find it hard to meet new people because of your vision loss. Social activities such as going to a restaurant can be logistically and emotionally challenging. This limits the enjoyment you feel from such events and the type of social events you can attend.
- Your visual impairment is not always obvious to other people which adds to the difficulties you experience.
- You sometimes require help from others to conduct your usual activities (e.g. shopping, cooking and paperwork).
- You can physically do most sports, such as gym work, especially with assistance. Your vision loss limits some of the types of sports you can take part in.
- Your eye condition does not cause you any pain.
- You require some support and adaptations to participate in work or education. Your condition means that some career paths and roles may not be an option for you.

**HS3 – LogMAR ≥0.6 and <1.0 or 20/80 to 20/200**

- You have an eye condition that has caused a moderate impairment of your central vision. You have some use of your peripheral vision. You have been told that your vision may continue to deteriorate.
- You are only able to read words on a page with a visual aid (e.g. magnification). You sometimes struggle to recognise people and may rely on a person’s shape/hair colour or voice in order to recognise them.
- You are able to use mobile phones, tablets and computers with adaptation (e.g. magnification).
- You can mostly navigate familiar environments with minimal difficulty. You have some difficulty navigating unfamiliar environments and may need assistance from another person or a visual aid.
- You are unable to drive and get limited use of public transport. Your visual impairment means that you rely on friends, family members, or taxis to take you where you need to go.
- Your vision loss can be very difficult to come to terms with. You often feel frustrated and worry about the future. You often feel anxious or depressed.
- You sometimes find socialising difficult. You often find it hard to meet new people because of your vision loss. Social activities such as going to a restaurant can be logistically and emotionally challenging. This limits the enjoyment you feel from such events and the type of social events you can attend.
- Your visual impairment is not always obvious to other people which adds to the difficulties you experience.
- You sometimes require help from others to conduct your usual activities (e.g. shopping, cooking and paperwork).
- You can physically do most sports, such as gym work, especially with assistance. Your vision loss limits some of the types of sports you can take part in.
- Your eye condition does not cause you any pain.
- You require some support and adaptations to participate in work or education. Your condition means that some career paths and roles may not be an option for you.

**HS4 – LogMAR ≥1.0 and <1.3 or 20/200 to 20/399**

- You have an eye condition that has caused a severe impairment of your central vision. You have some use of your peripheral vision. You have been told that your vision may continue to deteriorate.
- You are only able to read words on a page with a visual aid (e.g. magnification or read aloud technology). You rely on a person’s shape/hair colour or voice in order to recognise them.
- You get some use from devices like mobile phones, tablets and computers with adaptations (e.g. magnification or read aloud technology).
- You can mostly navigate familiar environments with minimal difficulty. You have a lot of difficulty navigating unfamiliar environments and often need assistance from another person or a visual aid.
- You are unable to drive and get limited use of public transport. Your visual impairment means that you rely on friends, family members, or taxis to take you where you need to go.
- Your vision loss can be very difficult to come to terms with. You often feel frustrated and worry about the future. You often feel anxious or depressed.
- You sometimes find socialising difficult and feel socially isolated. You find it hard to meet new people because of your vision loss. Social activities such as going to a restaurant can be logistically and emotionally challenging. This limits the enjoyment you feel from such events and the type of social events you can attend.
- Your visual impairment is not always obvious to other people which adds to the difficulties you experience.
- You often require help from others to conduct your usual activities (e.g. shopping, cooking and paperwork).
- You can physically do many sports, such as gym work, especially with assistance. Your vision loss limits the types of sports you can take part in.
- Your eye condition does not cause you any pain.
- You rely on support from others and adaptations to participate in work or education. Your condition means that some career paths and roles may not be an option for you.

**HS5 – LogMAR ≥1.3 and <1.7 or 20/399 to 20/1002**

- You have an eye condition that has caused a very severe impairment of your central vision. You have some use of your peripheral vision. You have been told that your vision may continue to deteriorate.
- You are only able to read words on a page with a visual aid. You can sometimes read words on a page using magnification but mostly use read aloud technology. You rely on a person’s shape/hair colour or voice in order to recognise them.
- You get some use from devices like mobile phones, tablets and computers with adaptations (e.g. read aloud technology).
- You can mostly navigate familiar environments with minimal difficulty. You have a lot of difficulty navigating unfamiliar environments and rely on assistance from another person or a visual aid.
- You are unable to drive and get limited use of public transport. Your visual impairment means that you rely on friends, family members, or taxis to take you where you need to go.
- Your vision loss can be very difficult to come to terms with. You often feel frustrated and worry about the future. You often feel anxious or depressed.
- You sometimes find socialising difficult and feel socially isolated. You find it hard to meet new people because of your vision loss. Social activities such as going to a restaurant can be very logistically and emotionally challenging. This limits the enjoyment you feel from such events and the type of social events you can attend.
- Your visual impairment is not always obvious to other people which adds to the difficulties you experience.
- You often require help from others to conduct your usual activities (e.g. shopping, cooking and paperwork).
- You can physically do many sports, such as gym work, especially with assistance. Your vision loss limits the types of sports you can take part in.
- Your eye condition does not cause you any pain.
- You rely on support from others and adaptations to participate in work or education. Your condition means that some career paths and roles may not be an option for you.

**HS6 – counting fingers**

- You have an eye condition that has left you with almost complete central vision loss. You can count fingers held up in front of you. You have limited use of your peripheral vision. You have been told that your vision may continue to deteriorate.
- You cannot read words on a page and rely on read aloud technology. You can only recognise someone by their voice.
- You rely on adaptations (e.g. read aloud technology) to use electronic devices like mobile phones, tablets and computers.
- You can mostly navigate familiar environments with minimal difficulty. You are unable to navigate unfamiliar environments without the assistance of another person or visual aid.
- You are unable to drive and get limited use of public transport. Your vision loss means that you rely on friends, family members, or taxis to take you where you need to go.
- Your vision loss is devastating, and you find it very difficult to come to terms with. You often feel frustrated and worry about the future. You often feel anxious or depressed.
- You find socialising difficult and feel socially isolated. You find it hard to meet new people because of your vision loss. Social activities such as going to a restaurant can be very logistically and emotionally challenging. This limits the enjoyment you feel from such events and the type of social events you can attend.
- Your visual impairment is not always obvious to other people which adds to the difficulties you experience.
- You rely on others to help you conduct many of your usual activities (e.g. shopping, cooking and paperwork).
- You can physically do some sports, such as gym work, with assistance. Your vision loss limits the types of sports you can take part in.
- Your eye condition does not cause you any pain.
- You rely on support from others and adaptations to participate in work or education. Your condition means that some career paths and roles may not be an option for you.

**HS7 – hand motion**

- You have an eye condition that has left you with almost complete central vision loss. You are able to detect motion in front of you. You have very limited use of your peripheral vision. You have been told that your vision may continue to deteriorate.
- You cannot read words on a page and rely on read aloud technology. You can only recognise someone by their voice.
- You rely on adaptations (e.g. read aloud technology) to use electronic devices like mobile phones, tablets and computers.
- You can mostly navigate familiar environments with minimal difficulty. You are unable to navigate unfamiliar environments without the assistance of another person or visual aid.
- You are unable to drive and get limited use of public transport. Your vision loss means that you rely on friends, family members, or taxis to take you where you need to go.
- Your vision loss is devastating, and you find it very difficult to come to terms with. You often feel frustrated and worry about the future. You often feel anxious or depressed.
- You find socialising difficult and are socially isolated. You find it hard to meet new people because of your vision loss. Social activities such as going to a restaurant can be extremely logistically and emotionally challenging. This limits the enjoyment you feel from such events and the type of social events you can attend.
- Your visual impairment is not always obvious to other people which adds to the difficulties you experience.
- You rely on others to help you conduct most of your usual activities (e.g. shopping, cooking and paperwork).
- You can physically do some sports, such as gym work, with assistance. Your vision loss limits the types of sports you can take part in.
- Your eye condition does not cause you any pain.
- You rely on support from others and adaptations to participate in work or education. Your condition means that some career paths and roles may not be an option for you.

**HS8 – light perception**

- You have an eye condition that has left you with almost complete central vision loss. You are able to detect the difference between light and dark. You have very limited use of your peripheral vision.
- You cannot read words on a page and rely on read aloud technology. You can only recognise someone by their voice.
- You rely on adaptations (e.g. read aloud technology) to use electronic devices like mobile phones, tablets and computers.
- You can mostly navigate familiar environments with minimal difficulty. You are unable to navigate unfamiliar environments without the assistance of another person or visual aid.
- You are unable to drive and get limited use of public transport. Your vision loss means that you rely on friends, family members, or taxis to take you where you need to go.
- Your vision loss is devastating, and you find it very difficult to come to terms with. You often feel frustrated and worry about the future. You often feel anxious or depressed.
- You find socialising difficult and are socially isolated. You find it hard to meet new people because of your vision loss. Social activities such as going to a restaurant can be extremely logistically and emotionally challenging. This limits the enjoyment you feel from such events and the type of social events you can attend.
- Your visual impairment is not always obvious to other people which adds to the difficulties you experience.
- You rely on others to help you conduct most of your usual activities (e.g. shopping, cooking and paperwork).
- You can physically do some sports, such as gym work, with assistance. Your vision loss limits the types of sports you can take part in.
- Your eye condition does not cause you any pain.
- You rely on support from others and adaptations to participate in work or education. Your condition means that some career paths and roles may not be an option for you.
