# Supplementary material for: Leber hereditary optic neuropathy: utilities and carer burden from British and Irish participants
Source: Orphanet J Rare Dis. 2025 May 7;20:219. doi: 10.1186/s13023-025-03737-w (PMC12060539; doi:10.1186/s13023-025-03737-w)
Supplement: Supplementary file 3 — Additional file3 (DOCX 17 KB) [file 13023_2025_3737_MOESM3_ESM.docx]

**Supplementary file 3: Summary table describing the instruments and methods used to measure HRQL in the study**

| **Instrument or method used** | **Source** | **Description** | **Completion** | **Limitations** | **Reference** |
| --- | --- | --- | --- | --- | --- |
| EQ-5D-5L | EuroQol Group | A 5 item descriptive system which assesses:  Mobility/ Self care/ Usual activities/ Pain or discomfort/ Anxiety or Depression  Scored as preference weighted single index score  Also includes Visual Analogue Scale for self-rated health on 0-100 scale | Likert scale and rating scale  Less than 5 minutes to complete | Doesn’t specifically assess vision loss | *www.euroqol.org* |
| Health Utilities Index – 3 | Health Utilities Inc | HUI-3 classification system consists of 8 attributes including vision, hearing, speech, ambulation, dexterity, emotion, cognition, and pain. Also scored as a single index of health | Likert scale  Approximately 5-10 minutes to complete | Less widely accepted than EQ-5D-5L | *www.healthutilities.biz* |
| Time trade off | Published | A standard interview technique which asks participants to consider the relative value of health/ quality of life compared with length of life through choice tasks. At the indifference point the data imply the utility for a health state. | Structured interview  Takes 30-60 minutes | Complex to understand  May be subject to bias | *Torrance GW (1986),* J Health Economics*, 5, 1986 1-30,* |
| Visual analogue scale |  | A rating scale which ranges from 0 (dead or worst imaginable health) to 100 (full health or best imaginable health). This can be used to rate the value of health vignettes. | Rating scale for each health state  A few minutes to complete | Criticised for lacking validity and not being consistent with economic basis of decision making | *Torrance GW, et al. (2001)* Medical Decision Making *21(4):329-334.* |
| VFQ-25 | National Eye Institute | Measures the dimensions of self-reported vision-targeted health status that are most important for persons who have chronic eye diseases. | Likert scale  About 10 minutes to complete | Profile measure not suitable for utility estimation | [*https://www.nei.nih.gov/learn-about-eye-health/outreach-resources/outreach-materials/visual-function-questionnaire-25*](https://www.nei.nih.gov/learn-about-eye-health/outreach-resources/outreach-materials/visual-function-questionnaire-25) |
